# Bicuspid Aortic Valve Is Associated with Less Coronary Calcium and Coronary Artery Disease Burden

**DOI:** 10.3390/jcm10143070

**Published:** 2021-07-11

**Authors:** Gudrun Feuchtner, Sven Bleckwenn, Leon Stoessl, Fabian Plank, Christoph Beyer, Nikolaos Bonaros, Thomas Schachner, Thomas Senoner, Gerlig Widmann, Can Gollmann-Tepeköylü, Johannes Holfeld, Wolfgang Dichtl, Fabian Barbieri

**Affiliations:** 1 Department Radiology, Innsbruck Medical University, 6020 Innsbruck, Austria; Sven.Bleckwenn@gmx.de (S.B.); Gerlig.Widmann@i-med.ac.at (G.W.); 2 Department Cardiac Surgery, Innsbruck Medical University, 6020 Innsbruck, Austria; Leon.Stoessl@i-med.ac.at (L.S.); Nikolaos.Bonaros@i-med.ac.at (N.B.); Thomas.Schachner@i-med.ac.at (T.S.); Can.Gollmann-Tepekoeylue@i-med.ac.at (C.G.-T.); Johannes.Holfeld@i-med.ac.at (J.H.); 3 Department Internal Medicine III, Cardiology, Innsbruck Medical University, 6020 Innsbruck, Austria; Fabian.Plank@i-med.ac.at (F.P.); Christoph.Beyer@i-med.ac.at (C.B.); Thomas.Senoner@i-med.ac.at (T.S.); Wolfgang.Dichtl@i-med.ac.at (W.D.); 4 Department of Cardiology, Charite University Medicine, Campus Benjamin Franklin, 10117 Berlin, Germany

**Keywords:** bicuspid aortic valve, coronary calcium, coronary artery disease, computed tomography

## Abstract

(1) Background. Bicuspid aortic valve (BAV) is associated with genetic defects (NOTCH 1, GATA 5) and aortopathy. Differences in the flow patterns and a genetic predisposition could also affect coronary arteries. The objective was to assess the coronary artery calcium score (CACS) and coronary artery disease (CAD) burden by coronary computed tomography angiography (CTA) in patients with BAV stenosis, as compared to stenotic tricuspid aortic valves (TAV). (2) Methods. A retrospective case–control study. A total of 47 patients with BAV stenosis (68.9 years ± 12.9, 38.3% females) who underwent CTA were matched with 47 TAV stenosis patients for age, gender, smoking, arterial hypertension, dyslipidemia, diabetes, body-mass-index and chronic kidney disease. (3) Results. The coronary artery calcium score (CACS) was lower in BAV (237.4 vs. 1013.3AU; *p* < 0.001) than in TAV, and stenosis severity was less (CAD-RAD^TM^: *p* < 0.001). More patients with BAV had CACS zero (27.7% vs. 0%; *p* < 0.001). The majority (68.1%) of patients with BAV had no or non-obstructive CAD but only 25.5% of TAV (*p* < 0.001). Obstructive CAD (>50% stenosis) by CTA was more frequently observed in patients with TAV (68.1%; *p* < 0.001). (4) Conclusions and Relevance. Patients with BAV stenosis have markedly less coronary calcium and less severe coronary stenosis. CTA succeeds to rule out obstructive CAD in the majority of BAV, with adherent implications for TAVR planning.

## 1. Introduction

Bicuspid aortic valve (BAV) is a congenital malformation affecting 1–2% of the population [1], associated with aortopathy and genetic defects such as NOTCH1 [2] and GATA5 [3] mutations.

BAV-aortopathy is characterized by medial layer degeneration, loss of elastic fibers and smooth muscle cells, and extracellular matrix (ECM) degeneration [1]. Matrix metalloproteinases (MMP-2 and -9) are increased [4]. The clinical phenotypic variability indicates also epigenetic regulations related to valve-mediated hemodynamic flow disturbances on the aortic wall [5].

Aortic flow profiles are different in BAV [6] as compared to tricuspid aortic valves (TAV), with a rather helical than laminar flow pattern, due to the distinct BAV geometry with an O-shape and a narrower orifice. This geometry leads to higher flow velocities and increases aortic wall shear stress.

While BAV-aortopathy has been well explored, the coronary artery disease profile has not been investigated yet extensively. Due to distinct laminar flow profiles in the aortic root, the inflow speed of blood towards coronary artery ostia is different [6]. This could lead to differences in the coronary atherosclerosis profile.

Coronary CTA is a non-invasive imaging modality for the evaluation coronary artery disease (CAD) severity. The coronary artery calcium score (CACS) allows for quantification of total calcium burden. CTA is routinely performed in patients with severe aortic stenosis before transcatheter aortic valve replacement (TAVR) for procedure planning [7] for both TAV and BAV [8]. However, in more than 50% of patients with TAV stenosis, coronary CTA fails to exclude obstructive CAD due to high CACS [9].

Therefore, the purpose of this retrospective case–control cohort study was to investigate the differences in the CAD profile by CTA (stenosis severity) and CACS in patients with BAV as compared to TAV.

## 2. Materials and Methods

**Study design.** Patients with moderate-to-severe aortic stenosis (AS) referred for coronary CTA for planning of TAVR between 2009 and 2020, were screened. The study was designed as retrospective case–control trial.

**Exclusion criteria** were prior coronary artery bypass grafting and/or prior multiple stent implantation and TAV with secondary-degenerative fused leaflets.

Patients with congenital BAV were identified and matched for age, gender, the major CV risk factors and chronic kidney disease, with patients having TAV.

**Computed tomography (CT).** A non-enhanced prospective ECG-gated CACS scan with standardized scan parameters (detector collimation 64 × 1.5 mm; 120 kV) was performed. The Agatston Units (AU) was calculated [10]. Contrast-enhanced cardiac/aortoiliacal CTA was performed by using a 128-slice dual-source CT (Definition FLASH or DRIVE, Siemens Healthineers. Erlangen, Germany) with a detector collimation of 2 × 64 × 0.6 mm and a rotation time of 0.28 s. The aortoiliacal prospective ECG-synchronized CTA was triggered into the diastolic phase (70% of RR-interval).

In patients with normal kidney function, a retrospective ECG-gated cardiac scan was appended [8]. Both scans were triggered into arterial phase using bolus tracking (threshold of 100HU, ascending aorta) and by injecting an intravenous iodine contrast agent (*Iopromide, Ultravist 370*™, Bayer Healthcare, Berlin, Germany). Axial thin slice images were reconstructed with 0.75 mm slice width (increment, 0.4) for cardiac and 1 mm (increment, 0.7) for aorto-iliac CTA.

**CTA image analysis.** Curved multiplanar reformations (cMPR) of coronary arteries (LM, LAD, CX, RCA) using 3D post-processing software (SyngoVia^TM^, Siemens Healthineers, Erlangen, Germany) were generated. Coronary stenosis severity was scored according to CAD-RAD^TM^ (0–5) [11] as minimal <25% (1), mild 25–49.9% (2), intermediate 50–69.6% (3), severe 70%–99% (4) stenosis or 100% occlusion (5) and stent (6/S) on a per-coronary segment-base (AHA-modified-17-segment-classification).

**Statistical methods.** Statistical analysis was performed using SPSS™ software (V26.0, IBM SPSS Inc., Armonk, NY, USA).

Differences in continuous data between two groups were tested using the independent *t*-test or the Mann–Whitney-U-test (in case of non-normal data distribution such as the CACS), according to distribution. Distribution as assessed by inspection of histograms or the Kolomogorow–Smirnov test. Differences in categorical data were determined with Chi-Square and score data (CAD-RADS) with Kruskal–Wallis-test.

## 3. Results

Of 558 patients with aortic stenosis referred to CTA, 71 (12.7%) with BAV were identified.

After excluding patients with prior CABG, prior multiple stent implantations and aortic valves with secondary–degenerative fused leaflets (*n* = 24), 47 patients with BAV were included (38 (80.8%) with type 1 raphe R/L, 7 (14.9%) R/N, 1 (2.1%) L/N and 1 (2.1%) type 0). These patients were matched with 47 TAV patients for age, gender, smoking, arterial hypertension, dyslipidemia, diabetes and CKD (Table 1). Mean left ventricular ejection fraction (LVEF) was 56.8% ± 13.8 (range, 22–82). Mean transvalvular pressure gradient (PG) was 38.75 ± 14 mmHg (range, 14–71), and mean aortic valve area (AVA) 0.69 ± 0.16 cm^2^ (range, 0.49–1.0), respectively.

**Figure 1:**
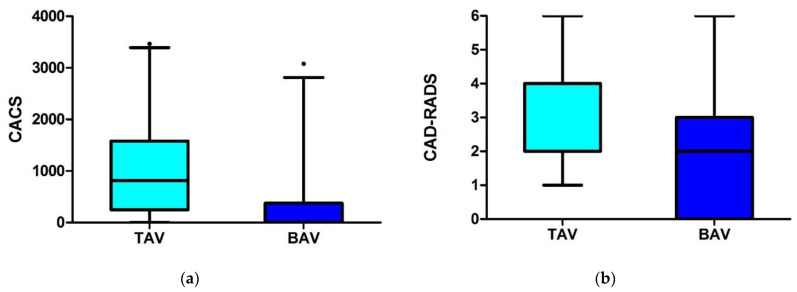
(**a**) Computed tomography (CT) findings (**a**) Coronary artery calcium score (CACS) in patients with bicuspid aortic valve (BAV) stenosis was significantly lower (*p* < 0.001) as compared to tricuspid aortic stenosis (TAV). (**b**) Coronary artery stenosis severity (CADRADS) in patients with bicuspid aortic valve (BAV) stenosis was lower (*p* < 0.001) as compared to tricuspid aortic stenosis (TAV). Dot = extreme values.

The coronary artery calcium score (CACS) was lower in BAV (237.4 vs. 1013.3 AU; *p* < 0.001), and stenosis severity score (CAD-RADS) by CTA was lower (*p* < 0.001) than in TAV (Figure 1, Table 2).

**Figure 2:**
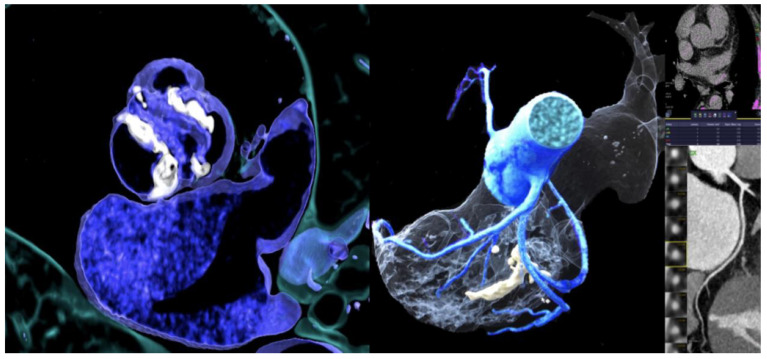
72 years-old-male with severe bicuspid AS (Type I, Raphe L/R) (left), zero CACS (right, upper) and no coronary stenosis by CTA (right).

The proportion of patients with CACS zero and no signs of CAD on CTA were higher in patients with BAV (27.7% vs. 0%, *p* < 0.001). The majority of patients with BAV had no or non-obstructive CAD (68.1%) (Table 2, Figure 2). Obstructive CAD (>50% stenosis) was more common in patients with TAV (68.1% vs. 25.5; p<0.001). Aortic valve calcium score (AVC) was not different between TAV and BAV patients (3024.7 ± 3102 vs. 3157.6 ± 2353 Agatston Units (AU), *p* = 0.379).

There were no coronary anomalies in the TAV group (0%), and 1 anomalous RCA arising from the opposite left coronary sinus in the BAV group (1/47; 0.2%).

## 4. Discussion

Surprisingly, the CACS and coronary stenosis severity by CTA were markedly lower in BAV as compared to TAV, despite carefully matching the patients for their CV risk profile.

The reasons could be 2-fold: either an unknown genetic predisposition related NOTCH1 or GATA 5 mutations [2,3] affecting the nitric-oxide (NO) synthase, or less wall sheer stress in the coronary arteries due to the distinct laminar flow profiles in the aortic root, with lower flow directed towards the coronary ostia in BAV [6].

In BAV, the narrower orifice and distinct O-shape leads to different flow profiles with higher flow velocity and wall sheer stress in the ascending aorta. In contrast, the flow speed and pressure directed towards the left and right coronary ostium is lower and may result in decreased wall sheer stress and consequently reduced coronary calcium load.

Remarkably, about one-quarter of elderly patients with BAV stenosis had zero CACS. This is very unusual for this age group, suggesting a genetic predisposition. In contrast, all patients with tricuspid valves had a positive CACS, and overall higher coronary calcium load and stenosis severity.

Similarly, the majority of patients with BAV had no or a non-obstructive CAD, but only one-quarter of patients with tricuspid AS.

Our study findings favor the use of CTA for exclusion of obstructive CAD in patients with severe AS and BAV scheduled for TAVR planning. This saves valuable time and contrast agent exposure in the diagnostic triage of patients. Prior studies have shown that CTA allows an exclusion of obstructive CAD in only about 40% of patients with severe AS scheduled for TAVR. These results are in line with our control group and with prior studies [9] having enrolled almost exclusively patients with tricuspid valves.

TAVR has recently emerged for treatment of BAV stenosis with promising initial results, despite technical challenges related to the distinct BAV geometry and coronary sinus asymmetry have resulted in higher complication rates [7]. Notably, in patients with BAV, CTA succeeded in the majority of patients (67.2%) to rule out coronary stenosis >50%.

Study limitations. We acknowledge the retrospective study design with potential inherent bias. Medication influencing coronary atherosclerosis such as statins and NOACs were not different, but more patients with tricuspid AS were taking ASA. Second, we did not compare the performance of CTA with invasive coronary angiography. CTA is prone to overestimate stenosis severity and >50% obstructive disease in the presence of severe coronary calcification [9], especially in patients with a CACS above > 400 Agatston Units.

## 5. Conclusions

Patients with BAV stenosis have lower CACS and less severe coronary stenosis. Obstructive CAD was ruled out by CTA in the majority of patients with BAV prior to TAVR, with adherent implications for TAVR planning. In the future, our study findings may incite further investigations on the underlying reasons.

## Figures and Tables

**Table 1:** Study population.

	BAV	TAV	*p*-Value
age	68.9 ± 12.9	70.0 ± 7.1	*p* = 0.461
gender	18 (38.3)	18. (38.3)	*p* = 1.000
smoking	8 (17)	8 (17)	*p* = 1.000
art HT	19 (61.7)	28 (59.6)	*p* = 0.999
dyslipidemia	26 (55.3)	30 (63.8)	*p* = 0.506
diabetes	13 (27.6)	14 (29.8)	*p* = 0.999
BMI	25.5 ± 5.4	27.5 ± 4.9	*p* = 0.054
Hyperuricemia	6 (12.8)	6 (12.8)	*p* = 1.000
AF	22 (46.8)	15 (31.9)	*p* = 0.195
COPD	11 (23.4)	8 (17)	*p* = 0.604
CKD	8(17)	6 (12.8)	*p* = 0.770
ASA 100 mg	19 (40.4)	33 (70.2)	*p* = 0.007
statins	25 (53.2)	26 (55.3)	*p* = 0.836
NOAC	9 (19.2)	12 (25.5)	*p* = 0.071

Abbreviations. AF = atrial fibrillation. Art HT = arterial hypertension. COPD = chronic obstructive pulmonary disease. CKD = chronic kidney disease. BMI = body mass index. NOAC = novel oral anticoagulants. ASA = Acetylsalicylic acid. Ordinal data are presented as counts *N* (%) and continuous data as mean +/- standard deviation (SD).

**Table 2:** Coronary artery calcium score (CACS) and coronary artery disease profile by CTA.

	BAV	TAV	*p*-Value
	*N* = 47	*N* = 47	
**CACS (AU)**			
mean	237.4	1013.3	
median (IQR)	9.1 (374)	814 (1330)	<0.001 *
**CAD-RADs**			
median (IQR)	2.0 (3)	4.0 (2)	<0.001 **
**CAD-RAD**			
0	13	0	
1	8	2	
2	11	10	<0.001
**no or <50% stenosis**	32 (68.1%)	12 (25.5%)	<0.001
3	5	5	
4 + 5	7	27	
**>50% stenosis**	12 (25.5%)	32 (68.1%)	<0.001

Abbreviations: CACS = coronary artery calcium score. AU = Agatston Units. CAD-RAD^TM^ = coronary stenosis severity score (0: no; 1: minimal <25% stenosis; 2: mild <50%; 3: moderate 50–69.9%. 4: severe 70–99%, 5: 100%; S/6: stent). * Mann–Whitney U test ** Kruskal–Wallis.

## Data Availability

Data are available on request but have not been shared in a public repository.
